# Disruptive e-prevention from 2019: dummy online drug selling sites to reach new consumers

**DOI:** 10.1093/eurpub/ckac130.057

**Published:** 2022-10-25

**Authors:** M Mau, C Bouchet-Mayer, S Bost, M Shelly, D Trauchessec

**Affiliations:** 1 e-prevention, PlaySafe, Paris, France; 2 Hospital “Outside the Walls”, Aremedia, Paris, France; 3 Santésih, Research Center in Sociology, University of Montpellier, Montpellier, France; 4 Fernand Widal Hospital, Assistance Publique–Hôpitaux de Paris, Paris, France

## Abstract

**Background:**

The last three decades have seen the development of chemsex, the diversification of substances through New Psychoactive Substances (NPS), and new technologies allowing people to buy online and find peers to consume on applications while remaining in private spheres (Trend, Cadet-Taillou, 2020). The latter has made it more difficult to reach users, so as to document their uses and the population, and to design public health schemes aimed at users, except through targeting metapopulations known to consume more than the general population (Léobon et al., 2018; Talley et al., 2011).

**Methods:**

The PlaySafe association designed two dummy websites, one selling GBL, the other NPS. These websites use the same graphics and syntactic codes as the main websites of the field, except for: 1) a fake drug named “love machine”; 2) an automatic redirection to a health promotion page instead of payment finalization. The information collected is: delivery region, birth year, gender, perceived usefulness of the health promotion messages, whether people would recommend to friends, the contents and quantities of the shopping cart, the time spent on each page, and data gathered via Google Analytics.

**Results:**

On both websites 21,459 order attempts have been placed. This pathfinder research project has allowed to reach 6,203 people on the GBL website in 30 months and 7,927 people in 12 months on the NPS website, with people spending on average 1 min 35 s on the first website's prevention page and 1 min 27 s on the second. Around 85% of people consider the content useful, among whom 75% would recommend the website to friends. This communication also aims to present the characteristics of the reached population.

**Conclusions:**

This innovative approach has allowed to precisely target a population escaping public health research and prevention schemes. It appears interesting to explore online prevention, especially since most of the respondents consider the experience helpful and recommendable.

**Key messages:**

- NTIC can serve research and actions in public health in order to reach online drug buyers.
- Monitoring the online drug market can be used to design actions by stakeholders.

